# Metavir and FIB-4 scores are associated with patient prognosis after curative hepatectomy in hepatitis B virus-related hepatocellular carcinoma: a retrospective cohort study at two centers in China

**DOI:** 10.18632/oncotarget.12152

**Published:** 2016-09-20

**Authors:** Rui Liao, Yi-Peng Fu, Ting Wang, Zhi-Gang Deng, De-Wei Li, Jia Fan, Jian Zhou, Gen-Sheng Feng, Shuang-Jian Qiu, Cheng-You Du

**Affiliations:** ^1^ Department of Hepatobiliary Surgery, the First Affiliated Hospital of Chongqing Medical University, Chongqing, China; ^2^ Liver Cancer Institute, Zhongshan Hospital, Fudan University, Shanghai, China; ^3^ Department of General Surgery, Mianyang Central Hospital, Mianyang, China; ^4^ Institute of Biomedical Sciences, Fudan University, Shanghai, China; ^5^ Department of Pathology and Division of Biological Sciences, University of California San Diego, La Jolla, California, USA

**Keywords:** liver cirrhosis, hepatitis B virus, cancer, surgery, prognosis

## Abstract

Although Metavir and Fibrosis-4 (FIB-4) scores are typically used to assess the severity of liver fibrosis, the relationship between these scores and patient outcome in hepatocellular carcinoma (HCC) is unclear. The aim of this study was to evaluate the prognostic value of the severity of hepatic fibrosis in HBV-related HCC patients after curative resection. We examined the prognostic roles of the Metavir and preoperative FIB-4 scores in 432 HBV-HCC patients who underwent curative resection at two different medical centers located in western (Chongqing) and eastern (Shanghai) China. In the testing set (*n* = 108), the Metavir, FIB-4, and combined Metavir/FIB-4 scores were predictive of overall survival (OS) and recurrence-free survival (RFS). Additionally, they were associated with several clinicopathologic variables. In the validation set (*n* = 324), the Metavir, FIB-4, and combined Metavir/FIB-4 scores were associated with poor prognosis in HCC patients after curative resection. Importantly, in the negative alpha-fetoprotein subgroup (≤ 20 ng/mL), the FIB-4 index (I vs. II) could discriminate between patient outcomes (high or low OS and RFS). Thus Metavir, preoperative FIB-4, and combined Metavir/FIB-4 scores are prognostic markers in HBV-HCC patients after curative hepatectomy.

## INTRODUCTION

Hepatocellular carcinoma (HCC) is a type of primary liver cancer that can result from chronic inflammation induced by hepatitis B or C virus (HBV or HCV) infection. The incidence of HCC has increased over the past decade, and it typically arises in the setting of liver cirrhosis [1, 2]. Despite the multiple treatment options for HCC and advances in surgical techniques, HCC patients with cirrhosis have a poor prognosis when diagnosed in a symptomatic phase [3]. Thus, reliable and convenient indicators are necessary in order to select optimal candidates for curative surgery and to predict patient prognosis.

Several risk factors for HCC have been identified previously. These include clinical characteristics (e.g. male sex, cirrhosis, elevated serum gamma-glutamyltransferase, and high HBV load) [4–7] and various molecular markers [8, 9]. HCC is characterized by a high frequency of fibrosis and cirrhosis, which may impact the local host inflammatory/immune microenvironment. In response to chronic injury induced by fibrosis or cirrhosis, inflammatory cells (e.g. hepatic stellate cells and macrophages) accumulate and can promote proliferation of premalignant cells and provide fertile ground for HCC development [10]. Additionally, unique inflammatory/immune response signatures derived from the remnant liver could affect HBV-associated HCC (HBV-HCC) patient outcomes [11, 12].

The severity of liver fibrosis (based on a histopathologic assessment after hepatectomy [13]) and the liver stiffness measurement (LSM, Fibroscan^®^) [14, 15] have both been associated with HCC recurrence and should be evaluated in patients with cirrhotic HCC. The Metavir liver biopsy histological staging system is frequently used to assess the severity of liver fibrosis. This scoring system could accurately distinguish between successive stages from normal liver (stage F0) to cirrhosis (F4) based on estimates of the transition rates during fibrosis progression [16–19]. Recently, several fibrosis staging scores and indices based on laboratory tests have also been used to assess hepatic fibrosis or cirrhosis. These scoring systems have been demonstrated to have high reproducibility and reliability [20–23]. In particular, the FIB-4 index showed a high degree of accuracy in predicting liver cirrhosis in both HBV and HCV patients [24, 25]. Interestingly, the FIB-4 score was shown to be an independent risk factor for HCC, which can lead to cirrhosis [26]. However, the prognostic role of the preoperative FIB-4 score in long-term HBV-HCC was likely underestimated. The aim of this study was to evaluate the influence of fibrosis using Metavir [19] and preoperative FIB-4 scores on HBV-HCC patient outcomes after curative resection. Our results provide a better understanding of the impact of chronic inflammation on HCC patient prognosis.

## RESULTS

### Baseline characteristics in the testing set

The baseline characteristics of the 108 HBV-HCC patients in the testing set are shown in Table 1. The median follow-up time was 24 months (range: 1-108 months). Among the 108 HBV-HCC patients, the mean tumor size was 6.2 ± 3.5 cm. According to the TNM staging system, 49.1% (53/108) of the patients in the study population had stage IIIA disease. There were no patients with Metavir fibrosis stage F0 (normal liver) in this study. A total of 67.6% (73/108) of the patients developed fibrosis (F1-3) and 32.4% (35/108) had histologically confirmed liver cirrhosis (F4). Several laboratory tests were correlated with the Metavir scores (Table 2). A negative correlation was observed between the Metavir scores and circulating platelet counts (r = −0.397, *P* < 0.001), whereas a positive correlation was observed between the international normal ratio (INR), creatinine, and aspartate aminotransferase (AST) positively and the Metavir scores (*r* = 0.342, 0.254, 0.274, and *P* = 0.008, *P* < 0.001, *P* = 0.004, respectively).

**Table 1:** Characteristics of patients in the testing and validation sets

Characteristics	Testing set (*n*= 108)	Validation set (*n*= 324)	*P*
Age	51±12	53±11	0.558
Gender (male)	85 (78.7%)	276 (85.2%)	0.116
ALT (U/L)	49.89±43.03	60.04±96.29	0.366
AST (U/L)	49.27±30.77	46.70±52.35	**0.012**
TB (mg/dL)	1.03±0.61	0.93±0.66	0.056
ALB (g/L)	40.81±6.63	43.53±4.67	**<0.001**
Creatinine (mg/dL)	0.78±0.19	0.91±0.52	**<0.001**
AFP (ng/ml)(>20)	60 (55.6%)	198 (61.1%)	0.493
HBV DNA level (>10^5^ copies/ml)	59 (54.6%)	195 (60.2%)	0.502
INR	1.07±0.15	1.03±0.10	**0.008**
Platelet count (10^9^/L)	140±66	144±63	0.573
Tumor number (single)	85 (78.7%)	283 (87.3%)	**0.041**
Vascular invasion (yes)	45 (41.7%)	84 (25.9%)	**<0.001**
Tumor encapsulation (yes)	22 (20.4%)	129 (39.8%)	**<0.001**
Tumor size (≤5.0cm)	48 (44.4%)	223 (68.8%)	**<0.001**
TNM stage (I-II)	55 (50.9%)	277 (85.5%)	**<0.001**
Postoperative TACE (yes)	50 (46.3%)	116 (35.8%)	0.052
Re-operation (yes)	12 (11.1%)	28 (8.6%)	0.444
Metavir score (F4)	35 (32.4%)	132 (40.7%)	0.124

Abbreviations: ALT: alanine aminotransferase; AST: aspartate *aminotransferase*; TB: *total* bilirubin; ALB: Albumin; AFP: alpha fetoprotein; INR: *international normal* ratio; TACE: transarterial chemoembolization

Cutoff value of categorical variable in the training and validation sets are as follows: Age: 51 and 53y; ALT: 50 and 60 U/L; AST: 49 and 47 U/L; TB: 1.03 and 0.93mg/dL; ALB: 41 and 44g/L; Creatinine: 0.78 and 0.91mg/dL; INR: 1.07 and 1.03; Platelet count: 140 and 144×10^9^/L.

**Table 2:** Comparison of patients by Metavir score

Characteristics	Testing Set (*n*= 108)			Validation Set (*n*= 324)		
	Non-cirrhosis (*n*= 73)	Cirrhosis (*n*= 35)	*P*	Non-cirrhosis (*n*= 192)	Cirrhosis (*n* = 132)	*P*
Age	50±11.75	54±11	0.121	52±12	54±11	0.163
Gender (male v female)	60 v 13	25 v 10	0.785	162 v 30	114 v 18	0.621
ALT (U/L)	46.93±39.66	56.06±49.39	0.344	58.66±91.54	62.06±103.14	**0.012**
AST (U/L)	42.79±25.24	62.77±36.78	**<0.001**	46.61±60.35	46.83±38.06	**0.002**
TB (mg/dL)	0.95±0.59	1.19±0.62	**0.009**	0.88±0.66	1.00±0.65	**0.039**
ALB (g/L)	41.51±6.84	39.37±5.99	0.102	44.39±4.61	42.29±4.50	**<0.001**
Creatinine (mg/dL)	0.74±0.17	0.86±0.21	**0.004**	0.90±0.55	0.94±0.47	0.452
AFP (ng/ml) (≤20 v >20)	31 v 42	17 v 18	0.552	70 v 122	56 v 76	0.280
HBV DNA level (≤10^5^ v >10^5^copies/ml)	34 v 39	15 v 20	0.837	79 v 113	50 v 82	0.566
INR	1.04±0.013	1.13±0.016	**0.005**	1.00±0.07	1.05±0.09	**<0.001**
Platelet count (10^9^/L)	156.22±61.69	106.66±63.70	**0.001**	157.59±58.77	124.89±64.82	**<0.001**
Tumor number (single v multiple)	55 v 18	30 v 5	0.220	172 v 20	111 v 21	0.145
Vascular invasion (yes v no)	28 v 45	20 v 15	0.067	54 v 138	30 v 102	0.277
Tumor encapsulation (yes v no)	17 v 56	5 v 30	0.279	108 v 84	68 v 64	0.401
Tumor size (≤5.0 v >5.0)	35 v 38	13 v 22	0.293	123 v 69	100 v 32	**0.028**
TNM stage (I-II v III A)	39 v 34	16 v 19	0.455	155 v 37	122 v 10	0.785
Postoperative TACE (yes v no)	30 v 43	15 v 20	0.621	62 v 130	54 v 78	0.767
Re-operation (yes v no)	6 v 67	5 v 30	0.469	16 v 176	12 v 120	0.812

Abbreviations: HBV: hepatitis B virus; HCC: hepatocellular carcinoma; ALT: alanine aminotransferase; AST: aspartate *aminotransferase*; TB: *total* bilirubin; ALB: Albumin; INR: *international normal* ratio; AFP: alpha fetoprotein; TACE: transarterial chemoembolization.

### Metavir and FIB-4 scores are correlated with clinicopathologic features and overall survival

**Figure 1:**
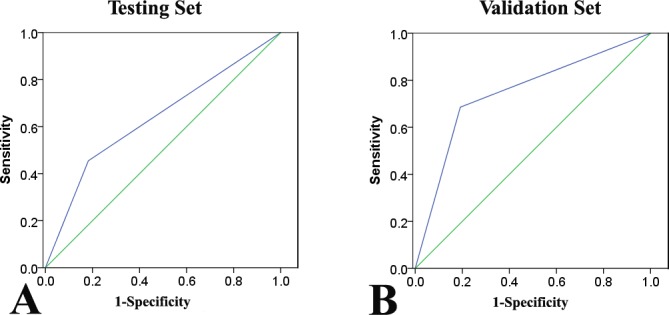
Receiver operating characteristic (ROC) curves of the FIB-4 index for prediction of cirrhosis according to the Metavir scores (Metavir F4) in the testing. A. and validation sets B

Metavir scores (non-cirrhosis vs. cirrhosis) were associated with several liver function-related laboratory tests such as AST, total bilirubin, creatinine, INR, and platelet count rather than tumor factors in the testing set (Table 2). The FIB-4 cutoff values were described previously [20]. Patients were classified into two subgroups at baseline: I: low scores (FIB-4 ≤ 3.25, *n* = 70) and II: high scores (FIB-4 > 3.25, *n* = 38). Similar to the Metavir scores, several clinicopathologic variables were associated with high FIB-4 scores (Table 3). We also found that the FIB-4 index was concordant with the Metavir score (r = 0.484, *P* < 0.001), while the FIB-4 (I and II) and Metavir scores (Metavir F1-3 and Metavir F4) were divided into two subgroups: non-cirrhosis and cirrhosis. A higher FIB-4 index (II) had a specificity of 89.8% and sensitivity of 54.5% for confirming the existence of significant cirrhosis (Metavir F4) with a positive predictive value of 83.2% (area under the receiver operating characteristic curve [AUROC]: 0.747; 95% confidence interval [CI]: 0.64-0.85, Figure 1).

**Table 3:** Comparison of patients by FIB-4

Characteristics	Testing Set (*n*= 108)			Validation Set (*n*= 324)		
	I (*n*= 70) ≤3.25	II (*n* = 38) >3.25	*P*	I (*n* = 229) ≤3.25	II (*n* = 95) >3.25	*P*
Age	48±10.8	57±10.3	0.180	51±11.2	57±10.3	0.102
Gender (male v female)	58 v 12	27 v 11	0.218	196 v 33	80 v 15	0.734
ALT (U/L)	45.76±35.88	57.05±53.51	0.409	49.71±45.35	64.33±110.57	0.917
AST (U/L)	41.04±17.84	64.42±42.20	0.248	36.85±20.18	70.45±87.31	**0.001**
TB (mg/dL)	0.89±0.44	1.28±0.77	0.391	0.84±0.35	1.14±1.05	**0.023**
ALB (g/L)	41.97±6.44	38.68±6.51	**0.043**	44.34±4.39	41.58±4.79	**0.003**
Creatinine (mg/dL)	0.76±0.16	0.80±0.23	0.452	0.91±0.52	0.92±0.52	0.221
AFP (ng/ml)(≤20 v >20)	30 v 40	18 v 20	0.652	91 v 138	35 v 60	0.707
HBV DNA level (≤10^5^ v >10^5^copies/ml)	34 v 36	15 v 23	0.421	91 v 138	38 v 57	1.000
INR	1.02±0.01	1.14±0.03	0.051	1.00±0.07	1.07±0.10	**0.002**
Platelet count (10^9^/L)	167.71±54.85	89.39±54.87	**<0.001**	167.00±57.60	89.47±37.76	**0.009**
Tumor number (single v multiple)	53 v 17	32 v 6	0.337	196 v 33	87 v 8	0.198
Vascular invasion (yes v no)	24 v 46	24 v 14	**0.005**	58 v 171	26 v 69	0.404
Tumor encapsulation (yes v no)	17 v 53	5 v 33	0.215	122 v 107	54 v 41	0.624
Tumor size (≤5.0 v >5.0)	34 v 36	14 v 24	0.311	154 v 75	69 v 26	0.360
TNM stage (I-II v III A)	40 v 30	15 v 23	0.107	197 v 32	80 v 15	0.729
Postoperative TACE (yes v no)	30 v 40	15 v 23	0.332	74 v 155	42 v 53	0.417
Re-operation (yes v no)	7 v 63	4 v 34	1.000	15 v 214	13 v 82	0.803

Abbreviations: HBV: hepatitis B virus; HCC: hepatocellular carcinoma; ALT: alanine aminotransferase; AST: aspartate *aminotransferase*; TB: *total* bilirubin; ALB: Albumin; INR: *international normal* ratio; AFP: alpha fetoprotein; TACE: transarterial chemoembolization.

**Figure 2:**
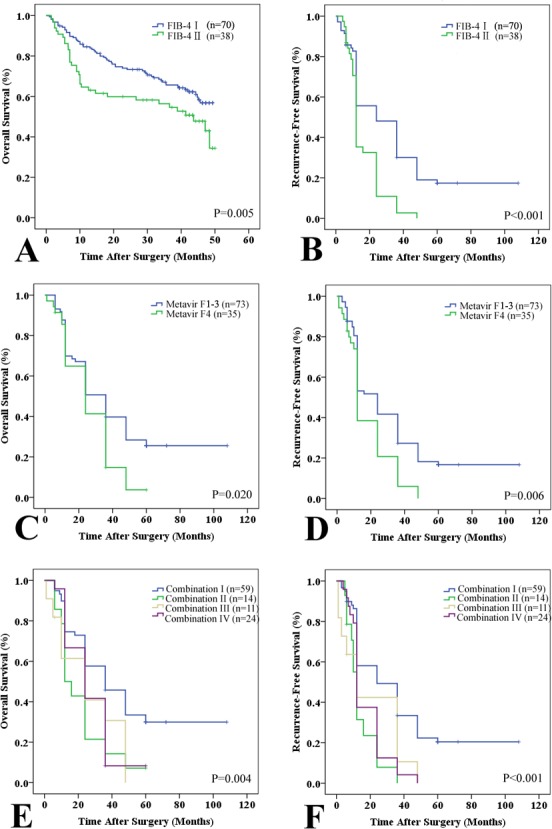
Prognostic value of the FIB-4 and Metavir scores in the testing set Patients were divided into two subgroups according to the FIB-4 and Metavir scores: low (Metavir F1-3 or FIB-4 I ≤ 3.25) and high (Metavir F4 or FIB-4 II > 3.25) scores. A and B: Kaplan-Meier plots of overall survival (OS, A) and recurrence-free survival (RFS, B) with low (FIB-4 I) and high FIB-4 (FIB-4 II) scores in the testing set. C and D: Kaplan-Meier plots of OS **C.** and RFS **D.** with low Metavir (F1-3) and high Metavir (F4) scores in the testing set. E and F: Kaplan-Meier estimates of OS **E.** and RFS **F.** stratified by the combined Metavir and FIB-4 scores (I: both low; II: low Metavir but high FIB-4; III: high Metavir but low FIB-4; and IV: high Metavir and FIB-4).

The 1-, 3-, and 5-year overall survival (OS) and recurrence-free survival (RFS) rates were 87.0%, 64.3%, 42.1%, and 78.3%, 51.4%, and 33.6%, respectively. On univariate analyses, albumin, tumor multiplicity, tumor encapsulation, tumor size, vascular invasion, TNM stage, and fibrosis stage (including the Metavir and FIB-4 scores) were correlated with OS and/or RFS (Table 4 and Figure 2). Multivariate analyses was performed on significant clinical factors. Tumor size, vascular invasion, tumor number, and FIB-4 score showed higher predictive value for OS and/or RFS.

**Table 4:** Prediction of survival and recurrence in the HBV-HCC population

Factors	OS				RFS			
	Univariate		Multivariate		Univariate		Multivariate	
	HR(95%CI)	*P*	HR(95%CI)	*P*	HR(95%CI)	*P*	HR(95%CI)	*P*
**Testing Set (n=108)**								
ALB (g/L) (≤41 v >41)	0.557(0.358-0.864)	**0.003**		0.061	0.612(0.402-0.933)	**0.007**		0.123
Tumor number (single v multiple)	1.795(1.093-2.946)	**0.008**	2.164(1.295-3.618)	**0.003**	1.467(0.900-2.391)	0.072		NA
Vascular invasion (yes v no)	0.440(0.285-0.680)	**<0.001**	0.580(0.370-0.909)	**0.018**	0.459(0.300-0.702)	**0.014**	0.596(0.381-0.933)	**0.024**
Tumor encapsulation (yes v no)	1.723(0.970-3.059)	**0.032**		0.461	1.476(0.879-2.477)	0.081		NA
Tumor size (≤5.0 v >5.0)	2.739(1.737-4.319)	**<0.001**	2.665(1.668-4.258)	**<0.001**	2.729(1.750-4.257)	**<0.001**	2.462(1.552-3.905)	**<0.001**
TNM stage (I-II v III A)	2.160(1.398-3.336)	**<0.001**		0.952	2.024(1.331-3.076)	**0.001**		0.618
Metavir score (F1-3/4)	1.590(1.019-2.481)	**0.020**		0.467	1.678(1.090-2.583)	**0.006**		0.920
FIB-4 (I/II)	1.751(1.125-2.727)	**0.005**	1.766(1.117-2.793)	**0.015**	2.061(1.330-3.195)	**<0.001**	2.057(1.308-3.236)	**0.002**
Combined M+F	2.984(1.672-3.784)	**<0.001**	3.027 (1.223-4.387)	**<0.001**	2.997(1.235-3.882)	**<0.001**	2.783(1.098-3.776)	**<0.001**
**Validation Set (n=324)**								
ALB (g/L) (≤44 v >44)	0.657(0.434-0.994)	**0.045**		0.474	0.838(0.608-1.156)	0.279		NA
TB (mg/dl) (≤0.93 v >0.93)	0.642(0.416-0.989)	**0.043**	0.619(0.398-0.962)	**0.033**	0.744(0.532-1.040)	0.082		NA
AST(U/L) (≤47 v >47)	1.759(1.174-2.635)	**0.006**		0.209	1.525(1.096-2.121)	**0.010**		0.343
AFP (ng/ml) (≤20 v >20)	1.530(1.001-2.337)	**0.048**		0.341	1.589(1.135-2.224)	**0.005**		0.174
Tumor number (single v multiple)	2.224(1.373-3.602)	**0.001**	2.563(1.568-4.188)	**<0.001**	1.728(1.140-2.620)	**0.008**	1.740(1.143-2.647)	**0.010**
Vascular invasion (yes v no)	2.016(1.342-3.027)	**0.001**	1.710(1.130-2.587)	**0.011**	2.164(1.561-3.000)	**<0.001**	1.933(1.380-2.708)	**<0.001**
Tumor size (≤5.0 v >5.0)	2.848(1.921-4.221)	**<0.001**	2.984(1.991-4.473)	**<0.001**	2.364(1.721-3.248)	**<0.001**	2.200(1.589-3.046)	**<0.001**
TNM stage (I-II v III A)	1.750(1.070-2.862)	**0.024**		0.108	1.758(1.178-2.623)	**0.004**	1.671(1.105-2.528)	**0.015**
Metavir score (F1-3/4)	1.602(1.082-2.373)	**0.018**	1.702(1.130-2.613)	**0.015**	1.377(1.005-1.886)	**0.045**		0.051
FIB-4 (I/II)	1.648(1.100-2.468)	**0.014**	1.662(1.071-2.579)	**0.023**	1.593(1.149-2.209)	**0.005**	1.663(1.197-2.312)	**0.002**
Combined M+F	3.232(1.893-5.434)	**<0.001**	3.332 (1.726-5.987)	**<0.001**	2.985(1.223-3.497)	**<0.001**	2.537(1.035-3.765)	**<0.001**

Univariate analysis: Kaplan-Meier method; multivariate analysis: Cox proportional hazards regression model. Abbreviations: OS: overall survival; TTR: time to recurrence; HBV: hepatitis B virus; HCC: hepatocellular carcinoma; ALB: Albumin; Combination M+F: combination of Metavir+FIB-4 scores; NA: not adopted.

The prognostic ability of combined FIB-4 (I/II) and Metavir (F13/F4) scores was reevaluated. Patients were divided into four groups: I: low Metavir and FIB-4 scores (*n* = 59); II: low Metavir but high FIB-4 scores (*n* = 14); III: high Metavir but low FIB-4 scores (*n* = 11); and IV: high Metavir and FIB-4 scores (*n* = 24). Significant discrepancies in OS (*P* = 0.004) and RFS (*P* < 0.001) were observed (low Metavir and FIB-4 scores vs. high Metavir and FIB-4 scores, Figure 1). Combined FIB-4 (I/II) and Metavir (F1-3/F4) scores predicted OS and RFS better than either alone (Figure S1 and Table S1). Based on the Metavir scores, non-cirrhosis (F1-3) was not correlated with either OS or RFS (*P* > 0.05). Recurrence was subdivided into early (≤ 24 months, *n* = 68) and late recurrence (> 24 months, *n* = 24). Univariate analyses indicated that patients with Metavir (F4) and FIB-4 (II) scores were more likely to exhibit late tumor recurrence.

**Figure 3:**
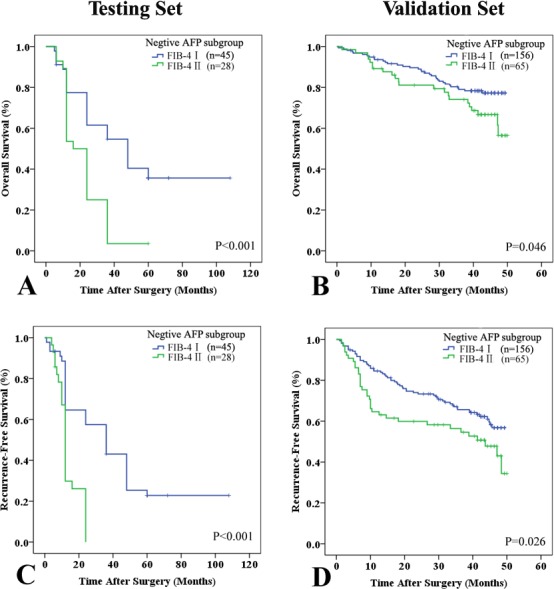
Kaplan-Meier analyses of the FIB-4 index in the negative alpha-fetoprotein (AFP) subgroup (u 20 ng/mL) All patients were classified into two subgroups: low (FIB-4 I ≤ 3.25) and high (FIB-4 II > 3.25) scores. In the negative AFP subgroup (F 20 ng/mL), the FIB-4 index could predict OS **A.** and **B.** and RFS **C.** and **D.** in the testing **A.** and **C.** and validation **B.** and **D.** sets.

We also found that several tumor factors were associated with early recurrence (Table 5). The prognostic significance of the FIB-4 score was also applicable to patients with negative AFP (≤ 20 ng/mL) in stratified analyses (Figure 3). To eliminate the influence of tumor factors, we assessed the impact of the FIB-4 score on tumor recurrence. Only patients with stage I or II HCC without vascular invasion were included in the analysis. Interestingly, we determined that the FIB-4 score could predict recurrence in these patients (Figure S2).

**Table 5:** Univariate and multivariate analyses for early and late recurrence

Factors	Testing Set				Validation Set			
	Univariate		Multivariate		Univariate		Multivariate	
	HR(95%CI)	*P*	HR(95%CI)	*P*	HR(95%CI)	*P*	HR(95%CI)	*P*
**Early recurrence**	*n* = 68				*N* = 115			
AFP	0.832 (0.487-1.419)	**0.038**		0.586		NA		NA
Vascular invasion	0.794 (0.472-1.337)	**<0.001**	0.233 (0.074-0.733)	**0.013**	0.479 (0.297-0.772)	**0.003**	0.479 (0.297-0.772)	**0.002**
Tumor number	1.645 (0.936-2.889)	**0.008**		0.065	1.815 (1.094-3.011)	**0.018**	1.815 (1.094-3.011)	**0.021**
TNM stage (I-II v IIIA)		NA		NA	1.735 (1.047-2.876)	**0.030**	1,735 (1.047-2.876)	**0.033**
**Later recurrence**	*n* = 24				n = 42			
ALB (≤41 v >41)	0.958 (0.346-2.658)	**0.028**		0.935		NA		NA
AST		NA		NA	0.581 (0.136-2.484)	**0.016**		0.595
FIB-4 (I/II)	2.816 (2.167-5.982)	**0.003**	3.035 (1.078-8.540)	**0.035**	1.969 (0.457-8.480)	**0.001**		0.466
Metavir score (F1-3/F4)	1.633 (0.507-5.261)	**0.021**		0.411	8.130 (2.183-30.276)	**<0.001**	8.699 (2.758-27.434)	**<0.001**

Univariate analysis: Kaplan-Meier method; multivariate analysis: Cox proportional hazards regression model.

Abbreviations: HR: Hazard Ratio; AFP: alpha fetoprotein; ALB: albumin; AST: aspartate *aminotransferase*; NA: not adopted.

### Association between FIB-4 scores and non-invasive markers

Non-invasive hepatic fibrosis markers such as the Forns index [27] and the aspartate aminotransferase-to-platelet ratio index (APRI) [21] can be used to predict HCC recurrence. Therefore, we compared the relationship between FIB-4 and other inflammation-associated parameters including the Forns index, APRI, Glasgow prognostic score (GPS), prognostic index (PI), and prognostic nutritional index (PNI) [28]. Our results indicated that the FIB-4 score was positively correlated with the Forns index and APRI in both sets (Table S2).

### Validation set

We next evaluated the predictive value of fibrosis stage in an additional series of 324 HBV-HCC patients from the Zhongshan Hospital of Fudan University (Table 1). The median follow-up time was 41.4 months (range: 1-50 months). The 1- and 3-year OS and RFS rates were 90.7% and 70.6%, and 77.1% and 54.1%, respectively. There were no statistically significant differences in the 1- and 3-year survival rates between the two sets (both *P* > 0.05). The mean tumor size at diagnosis was 5.1 ± 3.5 cm. According to the TNM staging system, 14.5% (47/324) of the study population had stage IIIA disease.

**Figure 4:**
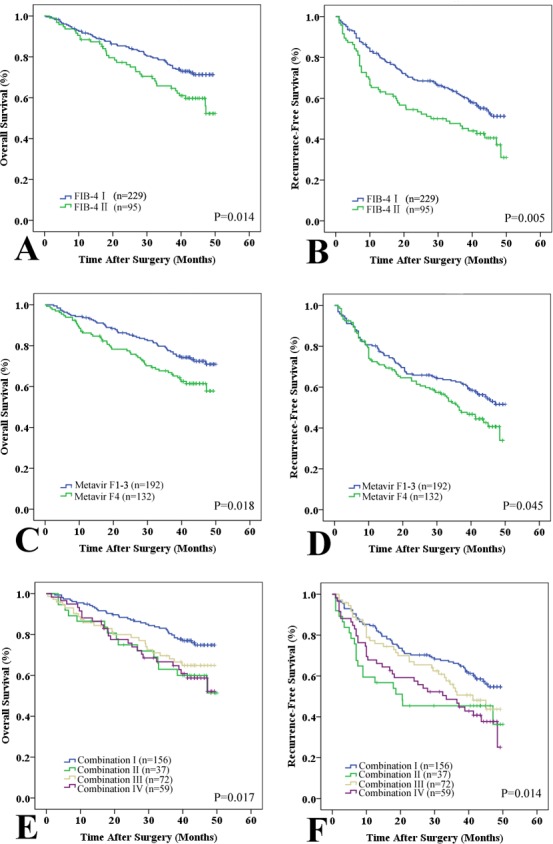
Prognostic value of the FIB-4 and Metavir scores in the validation set Patients were divided into two subgroups according to the FIB-4 and Metavir scores: low (Metavir F1-3 or FIB-4 I ≤ 3.25) and high (Metavir F4 or FIB-4 II > 3.25) scores. A and B: Kaplan-Meier plots of OS **A.** and RFS **B.** with low FIB-4 (I) and high (II) scores in the validation set. C and D: Kaplan-Meier plots of OS **C.** and RFS **D.** with low Metavir (F1-3) and high (F4) scores in the validation set. E and F: Kaplan-Meier estimates of OS **E.** and RFS **F.** stratified by the combined Metavir and FIB-4 scores (I: both low; II: low Metavir but high FIB-4; III: high Metavir but low FIB-4; and IV: high Metavir and FIB-4).

There were no significant differences in the mean fibrosis stage between the testing and validation sets. Similar to the results from the testing set, the FIB-4 index (FIB-4 I *vs*. II) was consistent with the Metavir score (Metavir F1-3 *vs*. F4, r = 0.294, *P* < 0.001). A higher FIB-4 index (II) had a positive predictive value for cirrhosis (Metavir F4) of 85.7%, with a specificity of 90.8% and a sensitivity of 31.4% (AUROC: 0.763; 95% CI: 0.57-0.70, Figure 1). The prognostic role of cirrhosis was validated in this independent cohort (Table 4). Univariate analysis revealed that cirrhosis (Metavir and FIB-4 scores) was significantly associated with poor OS (*P* = 0.018 and P = 0.014) and RFS (*P* = 0.045 and *P* = 0.005, Figure 4). Multivariate analysis also suggested that FIB-4 was a powerful prognostic marker for survival (HR = 1.662, 95% CI = 1.071-2.579, *P* = 0.023) and recurrence (HR = 1.663, 95% CI = 1.197-2.312, *P* = 0.002, Table S4). The prognostic ability of combined FIB-4 (I/II) and Metavir (F1-3/F4) scores was validated (Figure 4). Cirrhosis (defined by the Metavir and FIB-4 scores) was correlated with late recurrence (Table 5). Similarly, the FIB-4 index also showed prognostic value in patients with negative AFP (Figure 3).

## DISCUSSION

Although the outcomes of the majority of HCC patients are closely associated with cancer and cirrhosis, the potential contribution of cirrhosis to post-operative recurrence has not been adequately evaluated. Several clinical studies have demonstrated that the Ishak stage [13] and LSM [14, 15, 29] are useful predictors of HCC prognosis. In this study, we used pre-operative serum biochemical markers to assess the severity of liver fibrosis based on the FIB-4 index. We found that Metavir, preoperative FIB-4 scores, and combined Metavir and FIB-4 scores were associated with worse outcomes in HBV-HCC patients after hepatectomy. The FIB-4 index was utilized because it can predict liver cirrhosis in HBV and HCV patients [24, 25]. Since the FIB-4 index can be expressed as a continuous value, it may be used to evaluate the progression and severity of liver fibrosis. The FIB-4 score was also independently associated with HCC risk. Recently, Toyoda et al. determined that the FIB-4 score could predict the outcomes of HCC patients (most of whom had HCV [62.7%]) after curative hepatic resection [30]. To understand the potential influence of fibrosis on recurrence in HBV-HCC patients, regardless of histological data, it is necessary to elucidate the association between FIB-4 scores and patient prognosis.

Our multivariate analysis revealed that cirrhosis (FIB-4 II), but not fibrosis (FIB-4 I), was associated with worse outcomes in HBV-HCC patients after curative hepatectomy. We speculate that inflammatory factors in the cirrhotic liver could contribute to this association. First, in most cases, surgical resection is targeted towards tumor rather than non-tumor tissue, and would be used to evaluate the risk of recurrence. HCC that develops in a cirrhotic liver constitutes an extremely heterogeneous inflammatory microenvironment, which is influenced by various tumor characteristics, liver function, and HBV activity. Second, cirrhotic tissues and active HBV activity in the liver remnant can enhance liver insufficiency and intrahepatic recurrence even after surgical reduction of the tumor burden. HCC relapse after curative therapy may be due to metastasis from *de novo* tumors that arise in the cirrhotic liver rather than the original tumor [31]. Third, the tumor can be seeded by circulating cancer cells (CTCs) after resection, which is a potential cause of local recurrence. Indeed, there is evidence [32] that CTCs are recruited to tumor sites by inflammatory cells derived from cirrhotic liver tissue. Finally, we confirmed that FIB-4 could predict tumor recurrence in patients with early stage (TNM I and II) HCC without vascular invasion. Therefore, we hypothesize that the Metavir and preoperative FIB-4 scores may reflect inflammation caused by cirrhosis and HBV activity, and subsequently liver injury. Regardless of the underlying mechanisms, suppression of chronic inflammatory activity in the liver remnant by systemic therapy may prevent recurrence.

We found that the FIB-4 score was associated with several fibrosis-related parameters in the testing set such as the albumin level (*P* = 0.043) and platelet count (*P* < 0.001). It was consistent with the data indicating that the FIB-4 score was associated with the Metavir score (r = 0.484, *P* < 0.001) in the testing set. Platelet count and the AST/alanine aminotransferase (ALT) ratio are components of the FIB-4 index, and are established predictors of liver cirrhosis [33, 34]. Nevertheless, conclusions regarding the fibrogenic roles of albumin and platelet count should be cautiously interpreted due to the possibility of extrahepatic disease. On the other hand, all patients in our analysis had HBV, and most exhibited a higher fibrosis stage (F2-3: *n* = 206/432) or cirrhosis (F4: *n* = 167/432), which were typical characteristics of an inflammatory microenvironment that could impact liver function. We hypothesized that long-term HBV infection may result in an inflammatory cascade that could contribute to liver injury, malignant transformation of chronically infected hepatocytes, and tumor growth [35, 36]. Hence, the preoperative FIB-4 score could reflect the inflammatory status of malignant liver tissue.

We found that patients with cirrhosis (defined by Metavir (F4) and FIB-4 (II) scores) were more likely to have late HCC recurrence, indicating inflammation could promote the dissemination of cancer cells. Based on these data, a combination of prognostic information (e.g. vascular invasion, tumor size, and tumor stage), Metavir, and FIB-4 scores may be useful for triaging patients who are at higher risk for HCC recurrence and metastasis following hepatic resection. AFP is a common serum marker that is used to screen HCC patients for recurrence. However, the clinical applications are debatable [37]. Some clinical data has suggested that it is difficult to monitor recurrence in HCC patients with normal AFP levels [38, 39]. Importantly, we found that the FIB-4 index had the ability to discriminate between patients with worse survival and higher recurrence rates even in the negative AFP subgroup (≤ 20 ng/mL). Therefore, patients with higher FIB-4 scores and negative AFP require closer follow-up since they have a higher risk of recurrence.

The same criteria for all subjective variables including laboratory results and tumor characteristics were used at both medical centers. Additionally, the histologic slides used to analyze fibrosis stage were re-evaluated by two independent liver pathologists who were blinded to the clinical outcomes. In this study, we only investigated HBV-HCC patients. The HBV genotype was not routinely examined in our department. Although the majority of patients received antiviral therapy (either lamivudine or entecavir), follow-up data were difficult to analyze because many patients had variable medication and/or discontinued treatment against physician advice. Therefore, the potential influence of antiviral therapy on the FIB-4 index and patient outcome is unclear. A prospective study to explore whether these results are applicable to HBV-HCC patients who did not undergo surgery following neoadjuvant or adjuvant therapy is in progress. Additional studies are required to evaluate this issue.

In conclusion, we have shown that a combination of Metavir and FIB-4 scores could predict HCC recurrence and unfavorable prognosis in HBV-HCC patients after curative resection. Our data may assist with triaging HBV-HCC patients who are at higher risk for recurrence following surgical resection, and with selecting more effective adjuvant treatment for HCC.

## MATERIALS AND METHODS

### Patients, follow-up, and post-operative histological evaluation

This retrospective cohort study was performed in accordance with the ethical guidelines of the 1975 Declaration of Helsinki and was approved by the Ethics Review Committee of the First Affiliated Hospital of Chongqing Medical University and the Zhongshan Hospital of Fudan University. Written informed consent was obtained from all patients. Between January 2004 and September 2009, 165 consecutive archived patient records were selected for analysis. The patients were pathologically confirmed to have HBV-HCC and were eligible for R0 resection at the First Affiliated Hospital of Chongqing Medical University. The inclusion criteria were the following: (1) tested positive for the HBV surface antigen and HBV DNA; (2) had valid and reliable laboratory test data; (3) had no preoperative extrahepatic metastases confirmed by computed tomography (CT) and/or magnetic resonance imaging (MRI); (4) did not receive any pre-operative anticancer treatments; (5) underwent complete resection of all tumor nodules; (6) had complete records and follow-up data including baseline characteristics, laboratory tests, imaging examinations, and continuous regular follow-up. There were 108 patients who qualified for the study and comprised the testing set. A total of 57 patients were excluded based on the above criteria.

A larger, independent cohort of patients was selected from the Zhongshan Hospital of Fudan University in 2007 (validation set, *n* = 324) using the same inclusion/exclusion criteria for validation studies. Patients were selected from two hospitals located in western (Chongqing) and eastern (Shanghai) China. Importantly, there is a higher incidence of HCC in eastern compared to western China. Epidemiological studies have shown that unhealthy dietary habits, living environments, and various carcinogenic factors can promote the development of HCC, which may explain the difference in HCC incidence between these two regions of China. In this study, we considered whether the predictive power of the FIB-4 index would be applicable in different regions of China. To certain extent, our data are representative of the Chinese population.

All patients had post-operative follow-up every month for the first 6 months after surgery, every 3 months between months 7-24, and every 6 months thereafter. Follow-up consisted of serum AFP measurement and abdominal CT or/and MRI examinations according to the postoperative time. The FIB-4 index (age x AST/platelet count [x 10^3^/μL] x [ALT]^1/2^) was calculated as described [20]. Briefly, a FIB-4 score ≤ 3.25 was indicative of mild or/and moderate fibrosis whereas a FIB-4 score > 3.25 was indicative of advanced fibrosis or cirrhosis. All blood samples were obtained two days before surgery.

After resection, liver specimens were assessed by two experienced hepatopathologists who were blinded to patient clinical information. The degree of fibrosis in peritumoral liver tissue (the distance of the surgical margin to the border of the resected tumor tissues was 1 cm) was evaluated using the Metavir system [19]. Liver fibrosis was divided into five levels according to the Metavir score: F0, normal; F1, portal fibrosis; F2, fibrosis with few septa; F3, numerous septa; and F4, cirrhosis. The TNM classification system of the International Union Against Cancer (7^th^ edition) and tumor characteristics such as size, capsule formation, and vascular invasion were assessed as described previously [12].

All patients were categorized based on the degree of fibrosis: mild or/and moderate fibrosis (FIB-4 ≤ 3.25 or Metavir scores: F1-3), and advanced fibrosis or cirrhosis (FIB-4 > 3.25 or Metavir score: F4). The FIB-4 results were compared with the Metavir scores. We also evaluated combined FIB-4 (I ≤ 3.25 and II > 3.25) and Metavir (F1-3 and F4) scores. Patients were divided into four groups: I: low Metavir and low FIB-4 scores; II: low Metavir but high FIB-4 scores; III: high Metavir but low FIB-4 scores; and IV: high Metavir and high FIB-4 scores.

### Statistical analysis

Continuous variables (e.g. age, ALT, AST, total bilirubin [TB], albumin [ALB], INR, and platelet count) were reported as the mean ± standard deviation (SD) and compared using Student's t tests or non-parametric Mann-Whitney U-tests. Categorical variables were expressed as a percentage and examined using χ2 or Fisher's exact tests. Correlations between variables were analyzed using Pearson's or Spearman ρ coefficient tests. The FIB-4 index was subdivided into two groups according to the cutoff values as previously described (I ≤ 3.25 and II > 3.25) [20]. Univariate analysis was performed using Kaplan-Meier survival estimates and compared using log-rank tests. Factors that were significant on univariate analysis (*P* < 0.05) were included in the multivariate analysis using a Cox proportional hazard regression model with a forward stepwise variable selection process to estimate OS and RFS. The AUROC was calculated in order to assess the diagnostic sensitivity and specificity of FIB-4 in differentiating between fibrosis (Metavir F1-3) and cirrhosis (Metavir F4). All statistical analyses were performed using SPSS 16.0 (SPSS, Inc., Chicago, IL, USA) and a two-tailed *P* < 0.05 was considered significant.

## SUPPLEMENTARY MATERIALS


