# Supplementary material for: Metavir and FIB-4 scores are associated with patient prognosis after curative hepatectomy in hepatitis B virus-related hepatocellular carcinoma: a retrospective cohort study at two centers in China
Source: Oncotarget. 2016 Sep 20;8(1):1774–87. doi: 10.18632/oncotarget.12152 (PMC5352096; doi:10.18632/oncotarget.12152)
Supplement: Supplementary file 1 [file oncotarget-08-1774-s001.pdf]

Metavir and FIB-4 scores are associated with patient prognosis after curative hepatectomy in hepatitis B virus-related hepatocellular carcinoma: a retrospective cohort study at two centers in China

Supplementary Materials

Table S1 The area under the receiver operating characteristic curve (AUROC) of Metavir score, FIB-4 and their combination

Characteristics		AUROC (95% CI)			
		Testing Cohort (n=108)		Validation Cohort (n=324)	
		RFS	OS	RFS	OS
Metavir score	(F1-3/4)	0.652 (0.528-0.779)	0.618 (0.496-0.740)	0.550 (0.499-0.624)	0.560(0.492-0.628)
FIB-4	(Ⅰ/ Ⅱ)	0.670 (0.548-0.792)	0.635 (0.515-0.755)	0.550 (0.487-0.612)	0.563 (0.494-0.632)
Combined M+F	both low<br>both high	0.708 (0.596-0.820)	0.669 (0.550-0.789)	0.573 (0.511-0.635)	0.583 (0.516-0.650)

Abbreviations: RFS: recurrence-free survival; OS: overall survival.

Table S2 Correlation between FIB-4 and other non-invasive markers

Parameters		FIB-4 (≤3.25 vs >3.25)					
		**Testing Cohort (n=108)**			**Validation Cohort (n=324)**		
		**n**	**r**	**p**	**n**	**r**	**P**
Forns index	≤ 6.9	65	0.581	**0.017**	213	0.325	**0.024**
	> 6.9	43			111		
APRI	≤1.5	55	0.313	**0.002**	204	0.216	**0.017**
	>1.5	53			120		
GPS	0	69	0.216	0.059	198	0.192	0.127
	1/2	39			126		
PI	0	61	0.192	0.173	189	0.257	0.335
	1/2	47			135		
PNI	0	71	0.201	0.097	204	0.362	0.213
	1	37			120		

Abbreviations: APRI: aspartate aminotransferase to platelet ratio index; GPS: Glasgow prognostic score; PI: prognostic index; PNI: prognostic nutritional index.

Figure S1

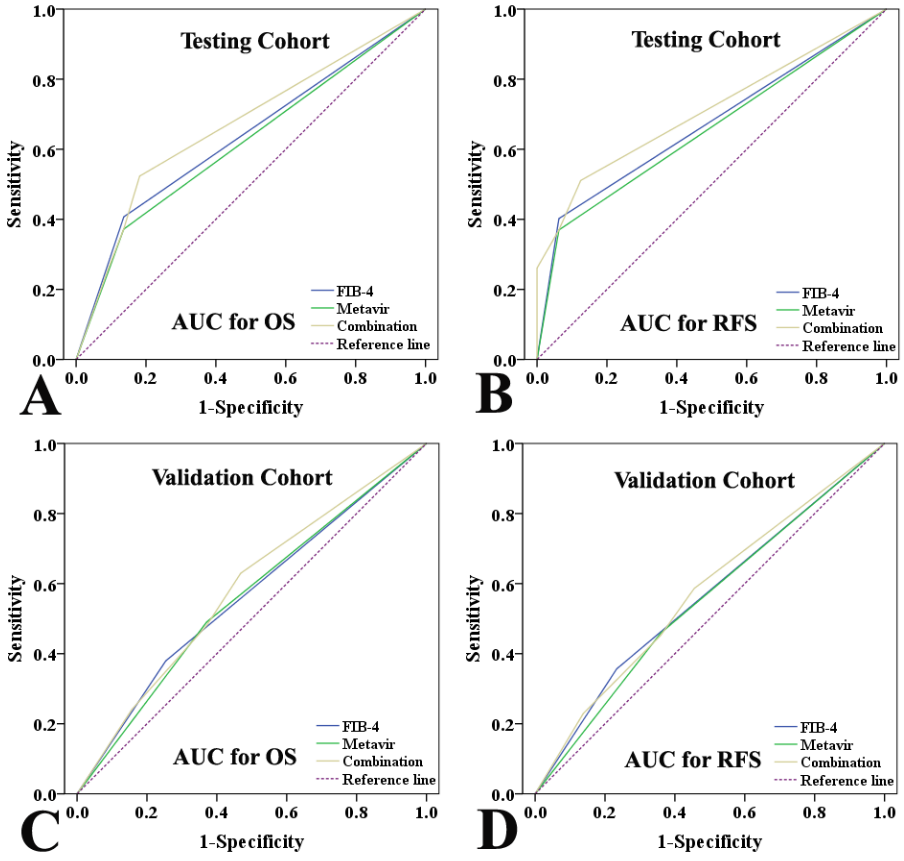

Figure S1. Receiver operating characteristics (ROC) curves of the predictive ability of the FIB-4, Metavir, and combined FIB-4 and Metavir scores in the testing (A and B) and validation sets (C and D).

Figure S2

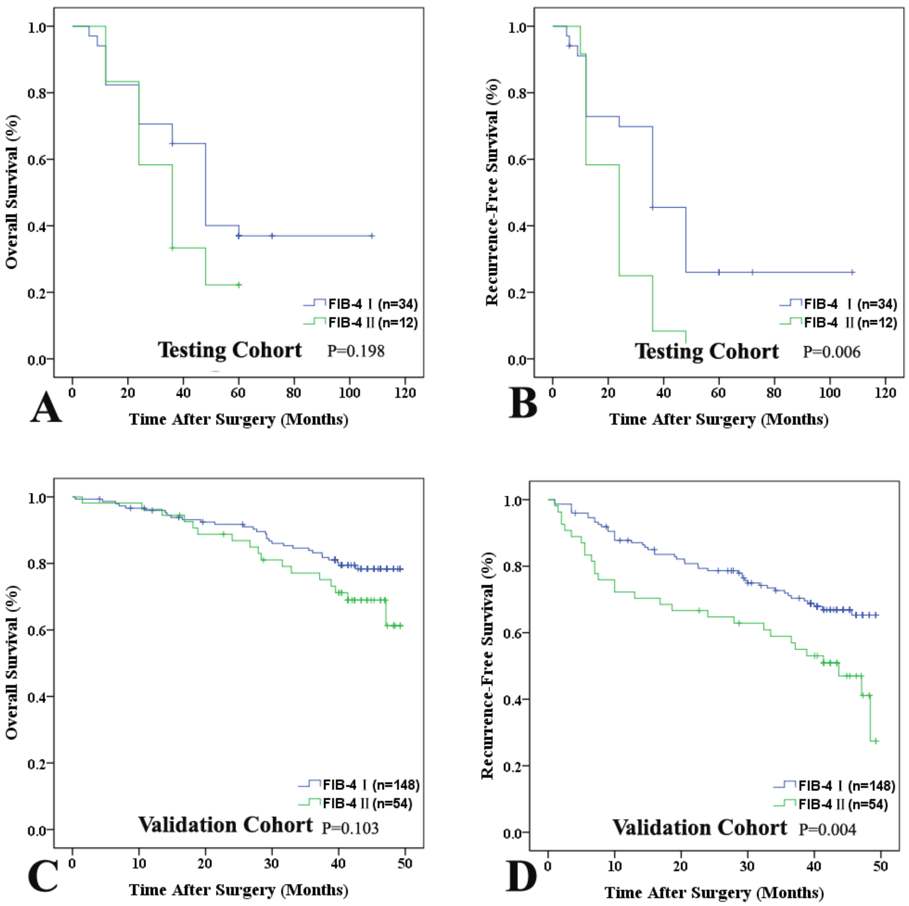

Figure S2. **Kaplan-Meier analyses of FIB-4 index in patients with early stage HCC (TNM I and II) without vascular invasion.** The FIB-4 index could predict RFS (C and D) and OS (A and B) in the testing (A and B) and validation (C and D) sets.
